# Encoding temporal information in deep convolution neural network

**DOI:** 10.3389/fnrgo.2024.1287794

**Published:** 2024-06-19

**Authors:** Avinash Kumar Singh, Luigi Bianchi

**Affiliations:** ^1^School of Computer Science, Faculty of Engineering and Information Technology, University of Technology Sydney, Sydney, NSW, Australia; ^2^Department of Civil Engineering and Computer Science Engineering, Tor Vergata University, Rome, Italy

**Keywords:** convolution neural network, electroencephalogram, temporal information, encoding, brain computer interaction (BCI)

## Abstract

A recent development in deep learning techniques has attracted attention to the decoding and classification of electroencephalogram (EEG) signals. Despite several efforts to utilize different features in EEG signals, a significant research challenge is using time-dependent features in combination with local and global features. Several attempts have been made to remodel the deep learning convolution neural networks (CNNs) to capture time-dependency information. These features are usually either handcrafted features, such as power ratios, or splitting data into smaller-sized windows related to specific properties, such as a peak at 300 ms. However, these approaches partially solve the problem but simultaneously hinder CNNs' capability to learn from unknown information that might be present in the data. Other approaches, like recurrent neural networks, are very suitable for learning time-dependent information from EEG signals in the presence of unrelated sequential data. To solve this, we have proposed an encoding kernel (EnK), a novel time-encoding approach, which uniquely introduces time decomposition information during the vertical convolution operation in CNNs. The encoded information lets CNNs learn time-dependent features in addition to local and global features. We performed extensive experiments on several EEG data sets—physical human-robot collaborations, P300 visual-evoked potentials, motor imagery, movement-related cortical potentials, and the Dataset for Emotion Analysis Using Physiological Signals. The EnK outperforms the state of the art with an up to 6.5% reduction in mean squared error (MSE) and a 9.5% improvement in F1-scores compared to the average for all data sets together compared to base models. These results support our approach and show a high potential to improve the performance of physiological and non-physiological data. Moreover, the EnK can be applied to virtually any deep learning architecture with minimal effort.

## 1 Introduction

Electroencephalogram (EEG) is widely used in research involving neural engineering, cognitive neuroscience, neurotechnology, and brain-computer interface (BCI). EEG signals are non-invasive, relatively cheaper to run, and provide high temporal resolution compared to other brain imaging techniques. However, EEG signals suffer from artifacts (eye, muscle, electrical noises, and broken sensors), non-stationarity, and inter- and intra-user variability. In a typical scenario, the researcher is required to process the acquired EEG signals to remove artifacts, extract features (time-frequency domain, spectrograms, and power ratios), and classify for specific tasks. No doubt, such work requires extensive domain knowledge and labor on top of the work needed to conduct an experiment and acquire EEG signals. Therefore, automating the whole process is essential, particularly with respect to real-time BCI applications such as diagnosis, supporting people with mobility disabilities, and entertainment. A recent development in deep learning techniques has attracted attention among EEG researchers, and the race has begun to develop a technique for better and more robust BCI. Despite several efforts to utilize different features of EEG signals in an automatic fashion, a significant research challenge is using unprocessed (raw) EEG data. It is to be noted that processed EEG data may or may not contain time-dependent information, which depends on the type of processing and the knowledge of the researcher. However, the raw EEG data naturally come with time-dependent features. Such features are highly crucial for decoding and classifying EEG signals.

**Figure 1:**
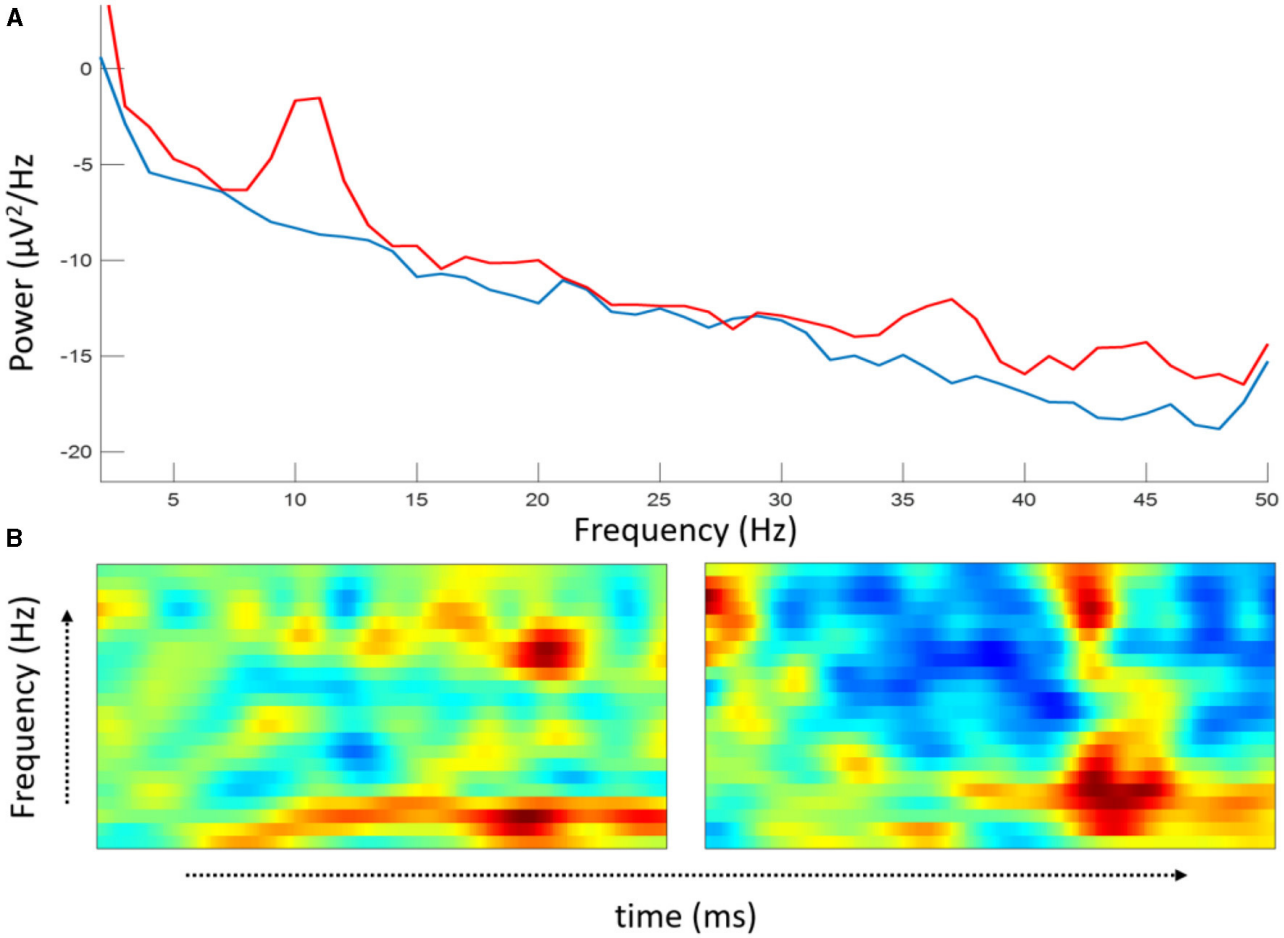
An example of time-dependent features of two EEG signals based on Aldini et al. (2019) at two different time points taken from the frontal region of the brain. **(A)** Power spectral density (PSD); **(B)** event-related spectral perturbation for PSD shown in blue **(left)** and red **(right)** signal.

Moreover, learning directly from time-dependent features diminishes the need for manual signal processing and feature extraction tasks and opens the possibility of extracting information previously unknown. An example of time-dependent features in the EEG signal is time-frequency information. Frequency information of an EEG signal alone can be seen as a feature. As shown in Figure 1A, the frequency of two signals is peaking at ~10 Hz, followed by similar but with smaller peaks at ~20 and 35 Hz due to the presented stimuli. What if we combined the frequency with time? To do that, we have converted two signals from Figure 1A into frequency over time (Figure 1B), also popularly known as an event-related spectral perturbation (ERSP) in the EEG community. The transformed information is full of features that cannot be reflected by the two signals alone in Figure 1A. This example clearly shows the importance of time-dependent features in EEG signals.

There has been significant research to EEG (Bashivan et al., 2015; Zhang et al., 2019; Li et al., 2020; Peng et al., 2024) to combine the time-dependency feature while learning local and global features. Despite that, the approaches are useful but often come with the cost of dedicated architecture to a specific task, are computationally expensive, and cannot be generalized to different tasks. This is another problem that hinders researchers' ability to apply developed models from one task to another. To solve these problems, we introduce the encoding kernel (EnK), a kernel-based approach for convolution operation. The EnK is a generalized approach to encode time information into the raw EEG signals while performing the horizontal convolution operation. Therefore, regardless of the task, the EnK creates a feature space for time-dependent information that is generalized to any architecture and interoperable with any EEG task. We have evaluated the efficacy and generability of the ENK with different EEG data sets collected from different tasks—cognitive conflict (Singh et al., 2018), physical human-robot collaboration (pHRC; Singh et al., 2020), P300 (Luck, 2014), and movement-related cortical potential (MRCP; Shibasaki et al., 1980). These data sets have been collected from different settings and environments and vary in the quality of signals, the number of EEG channels, the size of the data sets, and the number of participants. The main contributions of the EnK are as follows:

- The EnK is a novel approach to encode the time information in the data, with inspiration taken from traditional time-decomposition approaches for time series.
- The EnK does not require any domain-specific knowledge or handcrafted features; it therefore automates the time-dependent feature extraction process.
- The EnK is task-independent and architecture-independent; therefore, it can be applied to any new deep learning architectures.

### 1.1 Related work

The past few years have seen an increased number of deep learning applications in understanding and classifying EEG signals (Craik et al., 2019; Hossain et al., 2023). Deep learning has already shown a high number of successful applications in the field of natural language processing and computer vision, text classification, and action recognition (LeCun et al., 2015; Deng and Liu, 2018; Zhang et al., 2023).

A property of deep learning is to learn valuable information from raw data without manual labor (Chai et al., 2017), which is very useful in the case of EEG signals. Convolutional neural networks (CNNs) are one of the most popular methods in the field of deep learning and have proved effective in several EEG-based applications, such as epilepsy/seizures prediction (Emami et al., 2019; Lu and Triesch, 2019), the detection of visual-evoked responses (Du et al., 2023) and emotion recognition (Wang et al., 2023) and motor imagery (MI) classification (Tayeb et al., 2019; Dang et al., 2024).

Although deep learning can learn from raw EEG data, preprocessing of EEG signals is still required to reach optimal performance. These preprocessing methods highly depend on the type of data sets and expert knowledge such as filtering, channel referencing, and artifact removal methods. Furthermore, such preprocessed EEG signals also hinder deep learning models' ability to learn other relevant features, which might present in the data. A model that can learn from raw EEG data without handcrafted preprocessing and feature extraction is highly desirable, particularly for BCI applications.

There have been efforts to learn time-dependency information using CNNs. Liang and Hu (2015) utilized a recurrent CNN (RCNN) to learn dependencies in context and learn the context in neighboring information and showed an improved performance. Inspired by RCNN, Bashivan et al. (2015) trained EEG signals of mental workload using an RCNN. The authors showed that RCNNs could learn spatial, spectral, and temporal features from EEG signals and improve classification performance.

Cui et al. (2016) proposed a multiscale CNN (MCNN) model for time-series data. An MCNN automatically extracts features from identity mapping, downsampling, and spectrograms and locally convolve them. The convolved output is then followed by concatenation into a full convolution to predict time-series data. Lea et al. (2016) presented a temporal convolution model (TCN) that learned video-based action first by learning an individual frame of video using a CNN followed by an RNN. Although MCNNs and TCNs are promising approaches for capturing time-dependency features, they do not promise a similar performance for EEG signals.

Following the trend in the recurrent and convolution models, Zhang et al. (2019) proposed a convolution recurrent attention model (CRAM) for EEG signal analysis. A CRAM utilizes CNNs to learn high-level representation in EEG signals, while recurrent attention mechanisms are used to learn temporal features in EEG signals. The model showed a significant improvement in classifying the MI-based EEG signals. Recently, Li et al. (2020) proposed another approach based on a unified temporal-spectral features utilizing a group convolution squeeze-and-excitation network to detect epileptic seizures in EEG signals. Similarly, recently Ma et al. (2023) and Sharma et al. (2023) also presented a CNN model to learn from temporal-dependent features for motor imaging decoding and classification. It is claimed that this model can learn both spectral and temporal information from the epileptic seizure- and MI-based EEG signals but was only dedicated to specific EEG tasks. Therefore, it does not perform well for other EEG tasks or data sets.

Inspired by the progress in CNN models in the BCI community, Schirrmeister et al. (2017) proposed a very deep CNN model called DeepConvNet inspired from Visual Geometry Group (VGG) (Simonyan and Zisserman, 2014) for EEG signals. DeepConvNet demonstrated that it could learn different kinds of information related to EEG signals when decoding tasks. The model shows promising results, but it greatly depends on the size of input data to sufficiently converge and learn features.

Although several models have been proposed to learn time-dependency features primarily by combining recurrent and convolution networks, they are always dependent on the specific type of tasks to collect EEG signals. On the contrary, using a convolution network alone without any recurrent model has shown better success with decoding and classifying various tasks in EEG signals. Recently, there was a more generalized model demonstrated by Lawhern et al. (2018), called EEGNet. The EEGNet is focused on a compact CNN model utilizing the depthwise and separable CNN (Chollet, 2017) approaches. The EEGNet model has shown optimal performance from a variety of paradigms with EEG signals. The authors also showed that the EEGNet is very useful to generalizing on different EEG signals. Although the EEGNet is generalized for various EEG tasks, it does not utilize any specific deep learning module to learn temporal features. It is highly reliant on the CNN approach, which is well-known for learning local and global features only.

While the body of literature clearly shows an attempt to design and develop techniques to learn features like time-dependent information from EEG signals, they face the problems of (1) using fabricated and handcrafted features and (2) being highly customized or dedicated to very specific EEG tasks, for example, motor imagery (MI), emotions, event-related potentials (ERPs), and so on. It is to be noted that our goal is not to propose a new model or architecture of deep learning for EEG signal analysis. Instead, we have developed a novel technique to represent time-dependent information in EEG signals, which can be applied as a module to any existing deep learning model and significantly reduce the need for fabricated or handcrafted features while enhancing the applicability of approaches, that is, generalizing models for a variety of EEG tasks.

## 2 EnK: time-encoding approach

The EnK has been designed considering the classical theory of time decomposition (Das, 1994). The time-decomposition theory suggests that any given time-series signal comprises three components: a trend cycle, a seasonal component, and a remainder component. These components could be additive or multiplicative depending on the property of variance of signals. In general, EEG signals have high variance over time, but such variance in EEG signals can be reduced by slicing the data into smaller windows. The EnK also utilizes the smaller window of data at a time for convolution; therefore, a variance for such size was assumed to be zero. Following the time-decomposition theory for signals with no variance, we have considered an additive form of time decomposition rather than a multiplicative one. We have defined an EEG signal as comprising the following three major components.

### 2.1 Periodic component

EEG signals result from neuron excitation at different intervals, which creates periodic information. Most of this periodic information is in the combination of 0.1–100 Hz cycles, representing most brain activity in the delta (1–3.5 Hz), theta (3.5–8 Hz), alpha (8–13 Hz), beta (13–35 Hz), and gamma (35+ Hz) ranges related to any task including BCI (Deuschl, 1999).

To represent the periodic component in EEG signals, we have used sine functions (Vaswani et al., 2017). Consider EEG signals collected from *n* number of channels and defined as


(1) $$\begin{array}{l} X_{n}=x_{1},x_{2},x_{3},...,x_{n} \end{array}$$

Using the EEG signal represented by Equation (1), a periodic component can be represented as follows:


(2) $$\begin{array}{l} P_{t}=F\left(w_{t}*X_{n,t}+b_{t}\right), \end{array}$$

where F is sine function and *w*_*t*_ and *b*_*t*_ are weights and bias at time *t* for signal *X*.

### 2.2 Seasonal component

EEG signals have high temporal resolution, and it is challenging to evaluate seasonal variation for a short time recording like any other time series. However, EEG signals varied for inter- and intra-users. For example, intra-variability is due to EEG signals being recorded at different times of the day for different users, and inter-variability occurs when EEG signals are recorded in different mental states of the users.

The seasonal components are independent for each user and cannot be separated as an independent feature. It is required to learn for each user to generalize over a population. We have represented the seasonal component as follows:


(3) $$\begin{array}{l} S_{t}=\left(w_{t}*X_{n,t}+b_{t}\right)*P_{t}, \end{array}$$

where *P*_*t*_ is periodic component from Equation (2) and *w*_*t*_ and *b*_*t*_ are weights and bias at time *t* for signal *X*. *P*_*t*_ component as the multiplicative component is used to give the property of variance, which is not constant between periodic and seasonal components.

### 2.3 Artifacts

EEG signals suffer significantly from artifacts that arose from muscle, eye blinks, electrical inference, broken sensors, faulty equipment, and other unknown factors. These components usually distort the signals and reduce the signal to ratio. Most of these artifacts are found in very low frequency (<1 Hz) and high frequency (>45 Hz). One of the simplest and most effective methods used to reduce noise is filtering the EEG signals without distorting the phase. To do so, a band-pass filter is usually applied; however, it is well-known that a convolution also shares the property of band-pass filtering. Using convolution not only removes the artifact but also significantly reduces the complexity overhead from the EnK. Convolution has also been effective in similar practices as image as denoiser (Jain and Seung, 2008). In addition to the convolution filtering property, they also allow a weighted average of itself and its nearby neighbors' signals, which further reduces non-stationarity in the signals, therefore noisy values.

For EEG signal *X*, filtering and/or resampling using convolution can be defined as follows:


(4) $$\begin{array}{l} A=\overset{l}{\underset{j=1}{\sum }}\overset{m}{\underset{i=1}{\sum }}x_{i,j}*k_{i,j}, \end{array}$$

where *A* is artifact component and *x* is a signal filtered using kernel *k* with dimensions *l* and *m*.

### 2.4 EnK

Following the assumption that EEG comprises periodic, seasonal, and artifact components, we proposed the EnK, an approach to decompose the EEG signal into three components to encode time-dependent information better.

The periodic, seasonal, and artifact components of an EEG signal *X* at time *t* can be represented as


(5) $$\begin{array}{l} X_{t}=P_{t}+S_{t}+A_{t}, \end{array}$$

where the signals *P*_*t*_, *S*_*t*_, and *A*_*t*_ are the periodic (Equation 2), seasonal (Equation 3), and artifact components (Equation 4), respectively, at time *t*.

Using Equation (5), we can define **enk** as follows:


(6) $$\begin{array}{l} \text{enk}\left(X\right)=\{\begin{array}{cc} w*X_{n}+b, & \text{if}t=0,\\ P_{t}+S_{t}+A_{t}, & \text{if}0\leq t\leq k, \end{array} \end{array}$$

where *k* is the number of time points (samples) in a given EEG signal *X* with *n* number of channels.

## 3 Data description

We have used eight EEG data sets to evaluate our approach, comprising a mix of EEG tasks that generally induce features in the temporal, oscillatory, and temporal-oscillatory space. The data set description is shown in Table 1.

**Table 1:** Data description.

**Data sets**	**Channels**	**SR**	**Classes**	**Dimension**
P300	64	240	2	64 × 240 × 340
MI	22	250	2	22 × 750 × 1,296
pHRC	32	1,000	2	32 × 1,200 × 4,895
MRCP	28	1,000	4	28 × 500 × 316
DEAP	40	128	2	40 × 4,032 × 1,280

SR, sampling rate; P300, P300 visual-evoked potential; MI, motor imagery; pHRC, physical human-robot collaboration; MRCP, movement-related cortical potential; DEAP, Dataset for Emotion Analysis Using Physiological Signals.

### 3.1 pHRC

The cognitive conflict is an ERP elicited due to unexpected visual stimuli in EEG data. The visual stimuli are repeatedly presented to participants and asked to perform a certain task, and then a sudden change in expected behavior happens. Due to this, a negative deflection occurs 150–250 ms in the brain's frontal region, known as prediction error negativity, generally known to be found in the 4–13-Hz (theta and alpha) range. In the task, a participant performs the task in a real-world environment with ANBOT (Singh et al., 2020). The goal is to classify conflict with non-conflict conditions.

### 3.2 P300

The P300 is an ERP elicited due to visual stimuli in EEG data. The visual stimuli are based on an oddball visual paradigm. In this paradigm, participants were shown a non-frequent “target” with frequent “non-target.” The P300 waveform is a large positive deflection at ~250–350 ms on the parietal cortex whenever the target appears generally known to be found in the lower 01–4-Hz (delta and theta) frequency range. The EEG data used here have been taken from BCI Competition III (Dataset II; Blankertz et al., 2005). The goal here is to classify EEG signals into the target with non-targets.

### 3.3 MI and MRCP

Some neural activities contain both an ERP and an oscillatory component. One particular example of this is the MRCP, which can be elicited by voluntary movements of the hands, with features embedded in the 0.05–10-Hz frequency range (Jia et al., 2022, 2023). It is observable through EEG signals along the central and midline regions, contralateral to the hand or foot movement. The MRCP has been used previously to develop motor control BCIs for both healthy and physically disabled patients. The MRCP data used here are taken from BCI Competition II (Dataset IV; Blankertz et al., 2004). The goal here is to classify the four voluntary movements from hand and feet. Following similar oscillatory components due to imagined limb movement, we have also used other data from BCI Competition IV (Dataset 2a; Naeem et al., 2006). It contains four classes of limb movement. However, due to limited data, we have focused on two classification classes.

### 3.4 Dataset for Emotion Analysis Using Physiological Signals

The Dataset for Emotion Analysis Using Physiological Signals (DEAP; Koelstra et al., 2011) is based on EEG, Electromyography (EMG), respiration belt, plethysmograph, and temperature signals while participants are watching the 1-minute-long music video to induce four emotions (valences, arousals, like/dislike, and dominance). We have used the same dataset for different labels of four emotions (low/high). Therefore, four classification tasks were performed for each emotion.

## 4 Baseline methods

**Figure 2:**
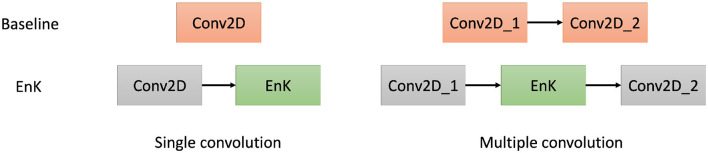
Illustration of uses of the encoding kernel with baseline models.

We have used four baseline models. These baseline models have been shown to generalize to different tasks in EEG signals with optimal performance in decoding and classification. The EnK approach has been used with these baseline models by adding a layer after the first convolution layer (see Figure 2).

1. EEGNet (Lawhern et al., 2018) is a compact CNN architecture and contains an input block, two convolutional blocks, and a classification block. EEGNet replaces the traditional convolution operation with a depthwise separable convolution inspired by Xception (Chollet, 2017).
2. DeepConvNet (Schirrmeister et al., 2017) is deeper and hence has many more parameters. It consists of four convolutional blocks and a classification block. The first convolutional block is specially designed to handle EEG inputs, and the other three are standard convolution ones.
3. ShallowConvNet (Schirrmeister et al., 2017) is a shallow version of DeepConvCNN, inspired by filter bank common spatial patterns. Its first block is similar to the first convolutional block of DeepConvNet, but with a larger kernel, a different activation function, a different pooling approach, and a classification block.
4. The RCNN, inspired by Liang and Hu (2015) contains five recurrent convolution layers to learn temporal features from the provided signals with a classification block.

### 4.1 Evaluation metrics and parameters settings

The performance of the EnK is compared to EEGNet, ShallowConvNet, DeepConvNet, and the RCNN. To compare, we have updated the same model with and without enabling the EnK layer and evaluated the mean squared error (MSE) and F1-score.

For the binary class, F1-score is calculated as absolute values, but for the multiclass, the F1-score is the weighted average for all labels. We have also compared the gradient-weighted class visualization map (Grad-CAM; Selvaraju et al., 2017) of the EnK after the first convolution with the first-layer output of the baseline models for P300, MI, pHRC, MRCP, and DEAP data sets (four conditions). It is noted that we have used the output of DeepConvNet only for comparisons.

For the statistical analysis, we used IBM SPSS (IBM Corporation, USA) to repeated measure analysis of covariance followed by a *post-hoc* analysis for paired comparison using a one-sample *t*-test.

The proposed model is fitted using the Adam optimization algorithm with default parameters as defined by Schirrmeister et al. (2017) and Lawhern et al. (2018). We ran a maximum of 200 training epochs with batch sizes ranging from 2 to 16 and dropout ranging from 15 to 75% for all data sets with early stopping after patience of 20 epochs. For all models, the learning rate was default set to be 0.001. The input data have been divided 60, 20, and 20%, respectively, for training, validation, and testing using stratified sampling. We trained the model with baseline models with the original structures and mostly original hyper-parameters and compared them with the enabled structure after the first convolution layer.

Each trained model has been saved together with the best weights, validation/training loss, and validation/training accuracy. All models were trained on a machine powered by NVIDIA Quadro P5000 Graphics Processing Unit, Org: Original (GPU), with CUDA 9 and cuDNN v7, and developed using Keras.^1^

## 5 Results and discussion

### 5.1 Performance comparison with and without EnK

As shown in Table 2, we have compared the effect of the EnK with standard EEGNet, ShallowConvNet, DeepConvNet, and RCNN models. The results clearly showed that the EnK approach significantly improves MSE, *F*(1, 22) = 20.326, *p* = 0.000, compared to the baseline models. In the *post-hoc* analysis to compare the paired models with and without EnK, it was found the EnK is significantly improved MSE from EEGNet (*p* = 0.030), ShallowConvNet (*p* = 0.045), DeepConvNet (*p* = 0.048), and RCNN (*p* = 0.026). However, as shown in Table 2, it can be seen the EnK does not always improve the RCNN, and in some cases, such as in the dominance and liking data sets, performance is comparable, while for P300 and MI, it is significantly low. One potential reason for the RCNN to work better than the EnK for some data sets is the ability to learn recurrent information from data sets over several layers. At the same time, the EnK module works only as one layer.

**Table 2:** The MSE EEGNet, ShallowConvNet, DeepConvNet, and RCNN with and without EnK after the first convolution for P300, MI, pHRC, MRCP, and DEAP (four categories) data sets. Except MRCP, all data sets have four classes.

	**EEGNet**		**ShallowConvNet**		**DeepConvNet**		**RCNN**	
**Data sets**	**Org**	**EnK**	**Org**	**EnK**	**Org**	**EnK**	**Org**	**EnK**
P300	0.074	**0.059**	0.074	**0.059**	0.059	**0.059**	**0.044**	0.500
MI	0.485	**0.369**	**0.231**	0.458	0.481	**0.431**	**0.192**	0.500
pHRC	**0.209**	0.328	**0.209**	0.377	0.209	**0.092**	0.209	**0.209**
MRCP	0.438	**0.438**	0.453	**0.422**	0.391	**0.344**	**0.422**	0.438
Valence	0.406	**0.244**	**0.430**	0.450	**0.402**	0.450	0.566	**0.434**
Arousal	0.410	**0.402**	0.410	**0.410**	0.410	**0.410**	0.590	**0.410**
Dominance	**0.371**	0.379	0.379	**0.379**	0.379	**0.379**	0.621	**0.621**
Liking	0.332	**0.332**	0.336	**0.324**	0.332	**0.328**	0.332	**0.332**
Mean ±*SD*	0.341± 0.14	**0.319** **±** **0.12**	**0.315** **±** **0.13**	0.36 ± 0.13	0.333 ± 0.14	**0.312** **±** **0.15**	**0.372** **±** **0.21**	0.43 ± 0.12

MSE, mean squared error; RCNN, recurrent neural network; Org,; Enk, encoding kernel; P300, P300 visual-evoked potential; MI, motor imagery; pHRC, physical human-robot collaboration; MRCP, movement-related cortical potential.; DEAP, Dataset for Emotion Analysis Using Physiological Signals. Best performance/value is highlighted in bold.

As a better measure, we also looked at the F1-score to better understand how well the EnK improved performance compared to baseline models. As it can be seen from Table 3 that the F1-score significantly increases, *F*(1, 22) = 28.421, *p* = 0.000), for the EnK compared to all baseline models. In the *post-hoc* analysis, it was found that the EnK significantly improved from EEGNet (*p* = 0.040) and ShallowConvNet (*p* = 0.006); however, there is an increase in F1-score but statistically not significant for DeepConvNet (*p* = 0.061) and RCNN (*p* = 0.056). It is to be noted that given that very few sets of data sets are used for comparison, there is always a possibility of not reaching enough statistical power to provide statistical significance, although there are differences.

**Table 3:** The F1-score from EEGNet, ShallowConvNet, DeepConvNet, and RCNN with/without EnK after the first convolution for P300, MI, pHRC, MRCP, and DEAP (four categories) data sets. Except MRCP, all data sets have four classes.

	**EEGNet**		**ShallowConvNet**		**DeepConvNet**		**RCNN**	
**Data sets**	**Org**	**EnK**	**Org**	**EnK**	**Org**	**EnK**	**Org**	**EnK**
P300	0.923	**0.941**	0.925	**0.941**	**0.941**	0.939	**0.956**	0.500
MI	**0.663**	0.662	**0.785**	0.667	0.638	**0.670**	**0.808**	0.500
pHRC	**0.883**	0.772	**0.883**	0.761	0.883	**0.925**	0.791	**0.791**
MRCP	**0.563**	0.546	0.544	**0.573**	0.601	**0.651**	**0.578**	0.563
Valence	0.409	**0.742**	0.587	**0.621**	0.605	**0.621**	0.434	**0.566**
Arousal	0.196	**0.400**	0.483	**0.582**	0.422	**0.582**	0.410	**0.590**
Dominance	**0.272**	0.199	0.271	**0.550**	0.229	**0.550**	0.379	**0.379**
Liking	0.078	**0.095**	0.271	**0.499**	**0.075**	0.023	0.668	**0.668**
Mean ±*SD*	0.498 ± 0.31	**0.545** **±** **0.29**	0.594 ± 0.25	**0.649** **±** **0.14**	0.549 ± 0.3	**0.62** **±** **0.28**	**0.628** **±** **0.21**	0.57 ± 0.12

RCNN, recurrent neural network; Org,; Enk, encoding kernel; P300, P300 visual-evoked potential; MI, motor imagery; pHRC, physical human-robot collaboration; MRCP, movement-related cortical potential; DEAP, Dataset for Emotion Analysis Using Physiological Signals. Best performance/value is highlighted in bold.

### 5.2 Grad-CAM data analysis

**Figure 3:**
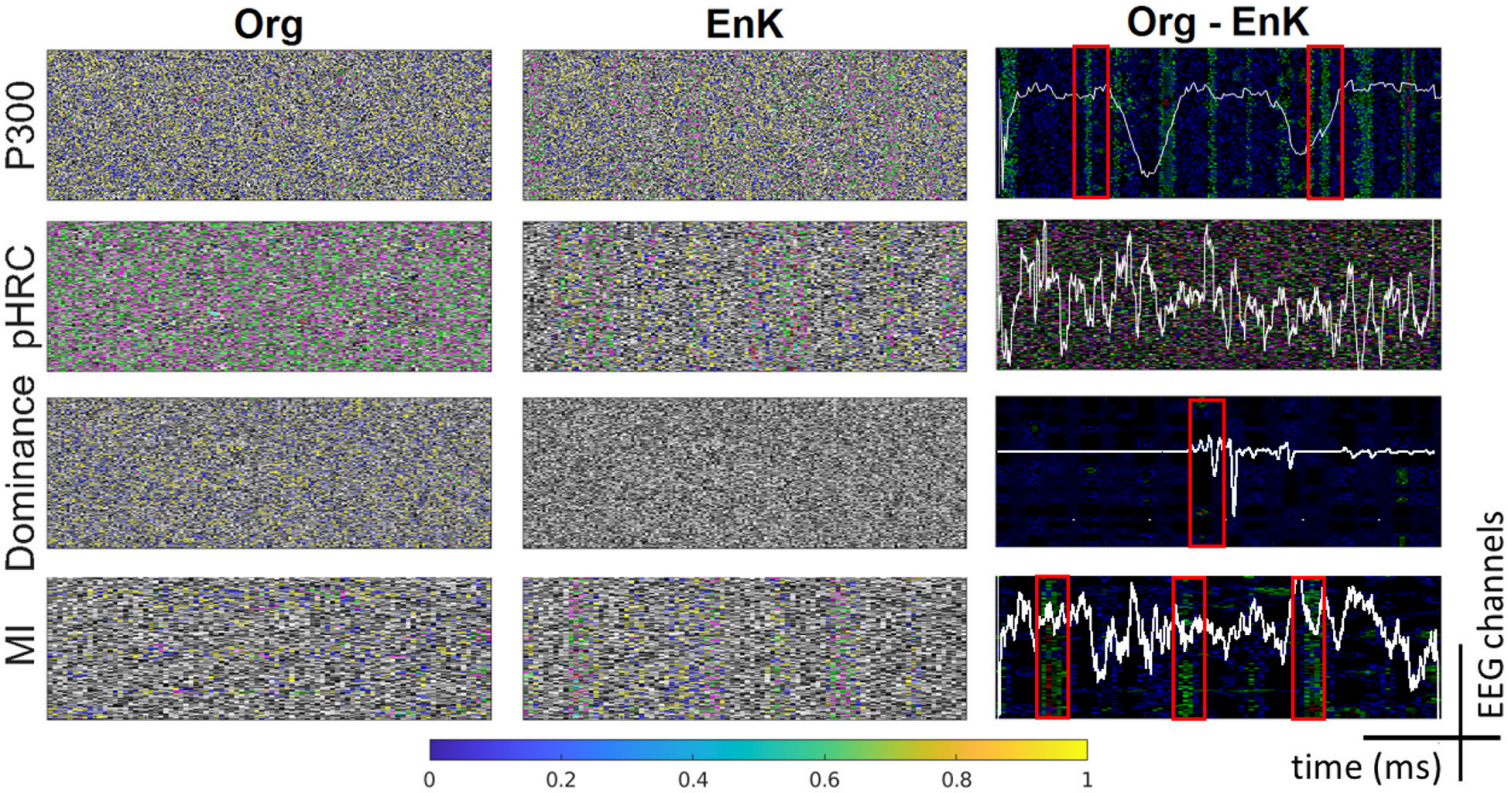
Grad-CAM from the original model **(first column)**, with EnK **(second column)**, and their difference **(third column)** for all datasets (P300, MI, pHRC, MCR, and DEAP data sets). The first and second columns are Grad-CAM output and therefore do not seem to have any value. However, the last column is overlayed with the original data (line graph) used to produce Grad-Cam results. The red box in the last column indicates the importance of the feature identified by the EnK, which is generally found in neuroscience literature. Grad-CAM, gradient-weighted class activation mapping; Enk, encoding kernel; P300, P300 visual-evoked potential; MI, motor imagery; pHRC, physical human-robot collaboration; MRCP, movement-related cortical potential; DEAP, Dataset for Emotion Analysis Using Physiological Signals.

Out of curiosity, to learn and understand the behavior of the EnK, we have also analyzed the gradient discovered for our data set compared to baseline models. For simplicity, the raw data (line graph from an EEG signal) has been overlayed in Figure 3 (last column). It can be seen from Figure 3 (last column) that EnK is successfully able to introduce time information in the data as assumed. This information can be seen as vertical lines representing the main features learned. For example, the last column for the P300 data set shows vertical lines, which, according according to Blankertz et al. (2005), represent a positive peak at 300 ms, that is, commonly known as P300 in the ERP data. This result clearly indicates that EnK can enhance P300 time-bounded information in data, which results in significant improvement in performance. Similarly, seeing the last column of MI data set shows that there are certain phenomena at several time points over the trial. All of them have been picked up by the EnK compared to the baseline models. However, the case of dominance and pHRC is an open question to be investigated from a neuroscience point of view. Something is happening that is highly related to the labeled information, but there is no clear literature to explain it. On the contrary, these gradient results could be used to further understand the phenomenon happening in the brain concerning the presented stimuli.

### 5.3 EnK approach and implications

These results clearly show that the EnK performs better than other comparable models, primarily due to its unique approach of encoding temporal information directly into the CNN architecture, as hypothesized. Existing CNNs typically focus on learning spatial and temporal features, but they struggle to extract information from time-dependent features, which are crucial for analyzing EEG signals. EnK addresses this limitation by decomposing the EEG signal into periodic, seasonal, and artifact components during the vertical convolution operation, enabling the CNN to learn both local and global time-dependent features effectively.

In addition to better performance, another significant advantage of the EnK is its ability to integrate temporal encoding into any existing CNN architecture without requiring domain-specific knowledge or handcrafted features. This generalizability makes it applicable across various EEG tasks and data sets.

The EnK could be applied in real-world scenarios, such as BCI applications, to significantly enhance performance. For example, in BCI applications for assistive technologies, the EnK's improved performance can lead to more reliable and efficient systems for people with disabilities. Additionally, the ability to automate the extraction of time-dependent features makes it more accessible and practical for broader use cases. The EnK has also shown potential as a tool for further investigating new or existing phenomena in cognitive neuroscience and potentially beyond.

## 6 Conclusion and future work

In this work, we have introduced the EnK approach, which encodes time information in CNNs. The EnK presents the data's time information by decomposing the signals into periodic, seasonal, and artifact components in an additive form before the model learns features. The EnK has been evaluated using various EEG data sets from different paradigms with varying sizes, channels, and sampling rates. Our results clearly show that the EnK is a promising approach compared to the state-of-the-art models because it leverages time-dependent features. In addition, the EnK shows a potential use case to explore new features and phenomena in EEG signals. Besides several advantages, the EnK can be used with any existing model and is independent of its architecture. We plan to introduce further refinements in the EnK approach and further explore various time-series signals in future work.

## Data availability statement

The original contributions presented in the study are included in the article/supplementary material, further inquiries can be directed to the corresponding author.

## Author contributions

AS: Writing - original draft, Writing - review & editing. LB: Writing - original draft, Writing - review & editing.

## Footnotes

^1^Keras: https://keras.io/.
